# TROPHIT1—a randomized, open-label, multicenter, phase II/III trial of sacituzumab govitecan compared to standard of care in metastatic colorectal cancer patients

**DOI:** 10.1016/j.esmogo.2024.100118

**Published:** 2025-01-17

**Authors:** B.C. Köhler, G.M. Haag, L. Le Cornet, P. Hoffmeister-Wittmann, M. Schmidt, A. Manjunath, N. Vaquero-Siguero, M. Jenzer, M. Gimmel, A. Stahler, A. Stein, M. Reichert, S. Kasper, M. Bitzer, D. Jäger, C. Springfeld, T.F. Weber, S. Fröhling, K. Steindorf, A. Trumpp, R.F. Schlenk, R. Jackstadt

**Affiliations:** 1 Department of Medical Oncology, National Center for Tumor Diseases, Heidelberg University Hospital, Heidelberg; 2 German Cancer Consortium (DKTK), DKFZ, Core Center Heidelberg, Heidelberg; 3 Clinical Cooperation Unit Applied Tumor-Immunity, German Cancer Research Center (DKFZ), Heidelberg; 4 NCT Trial Center, Heidelberg University Hospital and German Cancer Research Center (DKFZ), Heidelberg; 5 Division of Physical Activity, Prevention and Cancer, German Cancer Research Center (DKFZ) and National Center for Tumor Diseases (NCT) Heidelberg, Heidelberg; 6 Heidelberg Institute for Stem Cell Technology and Experimental Medicine (HI-STEM gGmbH), Heidelberg; 7 Cancer Progression and Metastasis Group, German Cancer Research Center (DKFZ) and DKFZ-ZMBH Alliance, Heidelberg; 8 Faculty of Biosciences, Heidelberg University, Heidelberg; 9 Charité—Universitätsmedizin Berlin, Berlin; 10 Hämatologisch Onkologische Praxis Eppendorf (HOPE), Hamburg; 11 TUM School of Medicine and Health, Department of Clinical Medicine—Clinical Department for Internal Medicine II, University Medical Center, Technical University of Munich, Munich; 12 Department of Medical Oncology, West German Cancer Center, University Hospital Essen, Essen; 13 University Hospital Tübingen, Tübingen; 14 Department of Radiology, Heidelberg University Hospital, Heidelberg; 15 Department of Translational Medical Oncology, National Center for Tumor Diseases, German Cancer Research Center (DKFZ), Heidelberg, Germany

**Keywords:** TROP2, clinical trial, resistance, patient-reported outcome, reverse translation

## Abstract

**Background:**

Colorectal cancer (CRC) ranks among the most common malignancies worldwide. Response rates to standard-of-care (SOC) treatment drop sharply beyond the second treatment line. Trophoblast cell surface antigen-2 (TROP2) acts in a plethora of cellular processes and ectopic expression is detected in a significant percentage of CRCs. Sacituzumab govitecan (SG) is composed of a TROP2-directed antibody armed with the topoisomerase inhibitor SN38. Thus, SG delivers SN38 to TROP2-expressing cancer cells. SG is approved for the treatment of metastatic breast cancer. Phase I/II data revealed a favorable safety profile and early signs of clinical activity in unselected metastatic CRC (mCRC).

**Patients and methods:**

TROPHIT1 is an open-label, randomized, multicenter, phase II/III trial to investigate the efficacy of SG in mCRC. Patients being refractory to ≥2 lines of prior therapy and an irinotecan-free interval of at least 6 months are enrolled. In the first part of the study, 20 patients are enrolled in the single-agent SG arm. Upon clinical efficacy in the first part, additional 60 patients are randomized (1 : 1) in the second part to single-agent SG compared with SOC. The primary endpoint is progression-free survival. TROPHIT1 contains a translational research program to unravel the determinants of resistance.

## Highlights

• TROPHIT1 is a phase II/III trial of sacituzumab govitecan (SG) in metastatic colorectal cancer.
• Patients are eligible beyond second line treatment and after a 6 months Irinotecan-free interval.
• Progression free survival (PFS) is the primary endpoint of TROPHIT1.
• Part I of TROPHIT1 enrolls 20 subjects in a single arm design.
• Part II compares SG to physician’s choice (1:1 randomized, 60 subjects), if part I demonstrates ≥2/20 objective responses and median PFS ≥4.3 months).
• TROPHIT1 is accompanied by an extensive translational research program.

## Graphical abstract

**Figure:**
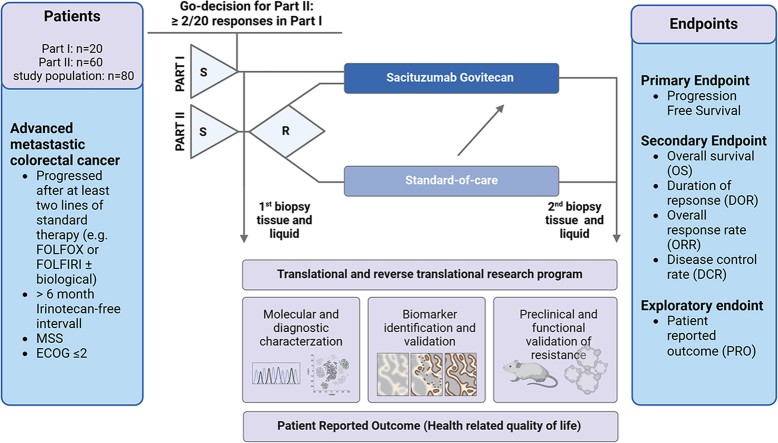

**Table:** Specifications table

Subject area	Medicine and dentistry
More specific subject area	Treatment of metastatic colorectal cancer
Name of your trial in progress	TROPHIT1—a randomized, open-label, multicenter, phase II/III trial of sacituzumab govitecan compared with standard of care in metastatic colorectal cancer patients
Reagents/tools	In part I and part II, sacituzumab govitecan is given intravenously at 10 mg/kg body weight on days 1 and 8 of a 21-day cycle. In part II, sacituzumab govitecan is compared with the physician’s choice in a 1 : 1 randomized manner.
Trial design	TROPHIT1 is a multicenter, randomized, open-label, phase II/III trial in Germany investigating sacituzumab govitecan in metastatic colorectal cancer (mCRC). Part I investigates sacituzumab govitecan in 20 individuals diagnosed with mCRC beyond the second line of standard treatment. Only if two or more responses are observed, and median progression-free survival (PFS) is at least 4.3 months, part II of the trial is initiated. Part II investigates sacituzumab govitecan compared with standard of care randomized 1 : 1. The primary endpoint is PFS.
Trial registration	NCT06243393, AIO-KRK-0124/ass
Ethics	The trial is conducted in accordance with the Declaration of Helsinki, the International Council for Harmonisation of Technical Requirements for Pharmaceuticals for Human Use (ICH) guidelines for Good Clinical Practice (GCP) and the European Union regulation 536/2014. Informed consent must be obtained from all participants before any clinical activity in this trial. Participants can withdraw their consent at any time. The trial was approved by the Paul Ehrlich Institute and the ethics committee on 19 April 2024.
Value of the trial in progress	There is an unmet medical need to develop specific, tolerable and effective therapies for mCRC. TROPHIT1 investigates sacituzumab govitecan, an antibody–drug conjugate (ADC) targeting trophoblast cell surface antigen-2 (TROP2), in mCRC beyond second line of treatment in individuals after a 6-month irinotecan-free interval. A translational research program unravels potential mechanisms of resistance to TROP2-directed ADC therapy in mCRC.

## Description of protocol

### Background

Worldwide >1 900 000 newly diagnosed colorectal cancer (CRC) cases are registered per year, many in incurable or relapsed stages.^1^ Treatment of metastatic CRC (mCRC) beyond second line remains challenging. Objective response rates are low and progression-free survival (PFS) is short. In patients treated with trifluridine/tipiracil and bevacizumab, considered the current standard in third-line treatment, only 6.1% of patients reach at least a partial remission, and the median PFS does not exceed 6 months.^2^ Re-challenge and re-exposure strategies utilizing conventional chemotherapies are commonly used but response rates remain low. There is a huge and evident medical need for more specific, less toxic and effective therapies.

Trophoblast cell surface antigen-2 (TROP2) transduces an intracellular calcium signal and is a cell surface receptor driving cellular processes such as proliferation. Membraneous TROP2 is expressed in various tissues including skin (keratinocytes), bladder epithelium (urothelium) and respiratory tract (alveolar and bronchial epithelium). The lower gastrointestinal tract and the mucosal epithelium of the colon express TROP2 on a very low level. Of note, TROP2 expression increases during colorectal carcinogenesis.^3^, ^4^, ^5^ Due to a high expression in various tumor types, therapeutic approaches directed against TROP2 were developed. Sacituzumab govitecan (SG) is an antibody–drug conjugate (ADC) linking a topoisomerase 1 inhibitor (SN-38) to a TROP2-directed antibody (hRS7). SG is Food and Drug Administration and European Medicines Agency approved for certain types of breast cancer. Importantly, phase III evidence and long-term results do not support a clear correlation between TROP2 expression and clinical response. For breast cancer, even tumors harboring a low TROP2 expression may respond to SG. However, the response is deeper and the clinical benefit is more pronounced in TROP2-high tumors.^6^^,^^7^ First preclinical evidence suggests that the majority of CRC cells show high TROP2 expression and might be dependent on TROP2-related functions making it a potential candidate target.

### Objective

TROPHIT1 aims to investigate the efficacy of SG in mCRC patients refractory to prior treatment and not eligible for local therapy. Individuals are eligible after at least two previous treatments according to standard of care (SOC) and, importantly, a minimum of 6 months of irinotecan-free interval. The primary endpoint is PFS.

Secondary endpoints are the assessment of (i) overall survival (OS), (ii) overall response rate (ORR, encompassing partial response and complete response), (iii) disease control rate and (iv) duration of response.

Exploratory endpoints assess (i) the change in patient-reported outcomes (PROs), including health-related quality of life (QoL) during treatment, and (ii) molecular characterizations, TROP2 expression and expression dynamics in patients <50 years of age.

**Figure 1:**
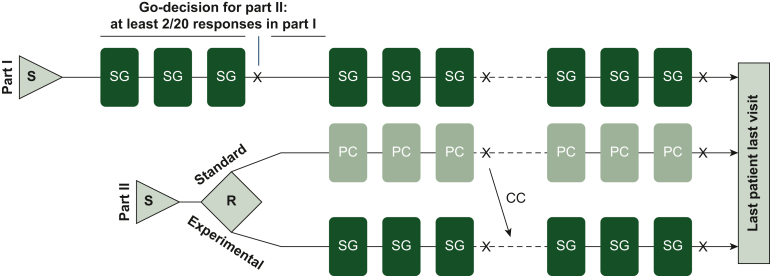
**Study design for part I and part II of TROPHIT1.** The initial first run-in part I enrolled 20 patients in a single-arm design (treatment with SG). In case of two objective responses and median PFS ≥4.3 months in part I, further 60 subjects will be enrolled in a randomized part II of the trial comparing SG with PC. Created with Biorender.com. CC, crossover; PC, physician’s choice; PFS, progression-free survival; R, randomization; S, screening; SG, sacituzumab govitecan.

The trial consists of two sequential parts. The first single-arm run-in part (part I) of the trial investigates efficacy assessed by PFS and objective response in 20 individuals. Part II will only be started if significant clinical efficacy and activity are observed in part I, defined as a median PFS time of ≥4.3 months and an ORR ≥10%. Subsequently, the randomized part II investigates the superiority of SG against SOC [physician’s choice (PC)]. Crossover to the experimental arm (SG) is allowed upon progression in the standard arm (PC) (Figure 1).

### Study design and statistical considerations

TROPHIT1 is a phase II/III, open-label, multicenter trial in Germany associated with the Arbeitsgemeinschaft Internistische Onkologie (AIO, AIO trial number AIO-KRK-0124ass). A phase II run-in part of the study is followed by a randomized phase III part. A design with a phase II run-in was chosen to allow an early evaluation of the clinical efficacy of SG in a limited number of patients with mCRC. After all patients enrolled in part I have completed their first three cycles or experienced an event, clinical efficacy is assessed. If no significant clinical efficacy (defined as a median PFS time of at least 4.3 months) and no activity (defined as ORR point estimate of ≥10%) can be shown, part II is not started. In case of efficacy (PFS ≥4.3 months) but no clear sign of activity (ORR <10%), a potential continuation of the trial will be discussed with the steering committee of TROPHIT1.

For part I, a two-sided, one-sample test using the maximum likelihood estimation of exponential data calculated from a sample of 19 subjects achieves 81.2% power at a 0.05 significance level to detect a median PFS time of 4.3 months with SG when the median PFS time of the historic control group is 2 months. For the randomized part II of the trial, a two-sided log-rank test with an overall sample size of 60 subjects (30 in the control group and 30 in the treatment group) achieves 80.1% power at a 0.05 significance level to detect a hazard ratio of 0.46512 when the control group median PFS time is 2 months. The study lasts approximately for 36 months of which subject accrual (entry) occurs in the first 24 months. The end of study (EOS) is defined as the date on which the last patient being alive and not yet withdrawn from study treatment and has been followed for 12 months.

The trial is activated in at least six cancer centers in Germany.

### Patient population

Patients diagnosed with mCRC are eligible after at least two lines of therapy. Trial inclusion criteria include a documented progression or intolerance to standard combinations for first- and second-line treatment (combinations) according to current guidelines including fluoropyrimidines, oxaliplatin and irinotecan. Previous epidermal growth factor receptor- and vascular endothelial growth factor receptor-directed therapies are allowed if indicated but not mandatory. Previous and preclinical data indicate a variety of mechanisms of transient and acquired resistance toward the topoisomerase inhibitors through prior topoisomerase exposure.^8^, ^9^, ^10^ Thus, a period of at least 6 months from the last administration of an irinotecan-based regimen is required.

A comprehensive list of inclusion and exclusion criteria can be accessed at https://clinicaltrials.gov/study/NCT06243393#participation-criteria. Inclusion criteria include:

• Irinotecan-free interval >6 months
• At least one measurable lesion according to RECIST 1.1
• Adequate bone marrow function
• Eastern Cooperative Oncology Group performance status ≤2

Phase III data in breast cancer (triple-negative breast cancer) led to an approval of SG irrespective of TROP2 expression status.^7^^,^^11^ It is possible that response to SG in mCRC is not necessarily dependent on high TROP2 expression on target cells. Thus, enrollment in TROPHIT1 is possible independently of and without prior assessment of TROP2 expression. However, TROP2 expression and comprehensive downstream analyses are carried out in the frame of TROPHIT1’s translational research program as outlined in the following section.

### Study trial treatment

All patients in part I and the experimental arm in part II receive SG 10 mg/kg on days 1 and 8 of a 21-day treatment cycle. Premedication consists of a histamine receptor 1 antagonist as well as steroids, and a 5-hydroxytryptamine-3 antagonist. Common toxicities for SG include neutropenia, gastrointestinal disorders and alopecia as the most frequently reported events. Most of the manageable toxicities may be attributed to the cytotoxic payload (SN-38).^6^^,^^7^ The use of prophylactic granulocyte colony-stimulating factor is not mandatory but allowed at the physician’s discretion if clinically indicated. Toxicities, which have been reported for other ADCs such as interstitial lung disease, are infrequently seen with SG.

Patients are treated until intolerable toxicity or progression.

Patients randomized to the control arm of part II receive one of the following SOC regimens as per physician’s discretion: regorafenib 160 mg on days 1-21 of a 28-day cycle, trifluridine/tipiracil 35 mg/m^2^ on days 1-5 and 8-12 of a 28-day cycle or trifluridine/tipiracil on days 1-5 and 8-12 of a 28-day cycle plus bevacizumab 5 mg/kg body weight on day 1 of a 14-day cycle.^2^^,^^12^^,^^13^ Dosing, dose adjustments and administration will be in accordance with approvals for any substances in the control arm. New drug approvals for advanced mCRC during the duration of TROPHIT1 may trigger an amendment.

### Tumor and safety assessment

Response evaluations are carried out based on computer tomography (CT) scans of the abdomen and chest every 9 weeks within SOC. Magnetic resonance imaging is an alternative modality for those subjects on treatment with a contraindication for CT scans at the physician’s discretion. The scans are evaluated centrally by trained radiologists at the University Hospital Heidelberg, Germany, according to the RECIST 1.1.

Adverse events (AEs) are graded according to the National Cancer Institute Common Terminology Criteria for Adverse Events (NCI CTCAE) v5.0. Recording of AEs is done in an electronic case report form in accordance with applicable regulations. Serious AEs and AEs of special interest have to be reported within 24 h.

## Trophit1 research program

### Translational and reverse translational research

TROPHIT1 is accompanied by a comprehensive research program. This research aims to understand the effect of SG treatment in mCRC on both molecular and clinical levels, to ultimately identify biomarkers to predict treatment response and define resistance mechanisms. To this end, we are utilizing biopsy material acquired to generate bulk or single-cell multiome (RNA and assay for transposase-accessible chromatin with high-throughput sequencing) as well as spatial transcriptomics data of metastases. Importantly, TROPHIT1 contains biopsies and blood samples at screening (SCR) and end of treatment (EOT).

Based on the transcriptome data, and in combination with TROP2 immunohistochemistry, we identify and characterize tumor subpopulations and assess their response to treatment. Of particular interest is the efficacy of SG in eradicating TROP2-positive populations and elucidating whether persisting cells can replenish the pool of TROP2-positive cells through cell state conversion. To address those questions, we make, for example, use of trajectory inference methods based on spliced and un-spliced RNA counts to be able to disentangle surviving from newly formed TROP2-positive cells. To better understand ultimate treatment resistance, we anticipate to identify distinct gene expression programs and gene regulatory networks that relate to the time points before and after SG-based treatment.

To extend the insights on tumor subpopulations to treatment response, patient-derived organoids are derived from the same biopsies, allowing to expand the patient tumor material in a dish while preserving cellular heterogeneity. Using correlation analysis, we link the clinical outcome to the molecular features such as TROP2 expression levels in the biopsies before treatment, changes in the abundance of tumor subpopulations, mutational profiles and the make-up of the microenvironment. Importantly, these patient avatars will enable us to define underlying mechanisms of therapy resistance and cell state transitions within a clinically meaningful context.

In addition, sequential liquid biopsy samples before the first sample and at progression are utilized to unravel SG-specific and ADC class-specific effects in a multilayer approach: multiplex protein biomarker analysis technology and circulating tumor DNA-derived shallow whole-genome sequencing are applied to identify and monitor traits of responding and non-responding individuals. Candidate targets that might confer treatment resistance to SG are experimentally validated in preclinical experiments.

### Patient-reported outcome research

Beyond response assessment during treatment, PROs may aid in measuring the potential magnitude of a clinical benefit through a specific intervention. Thus, TROPHIT1 contains questionnaire-based longitudinal PRO analyses as fundamental components of parts I and II of the trial. In addition to the overall health-related QoL assessment using the European Organisation for Research and Treatment of Cancer Quality of Life Questionnaire Core 30 (EORTC QLQ-C30) (summary score and function and symptom scores), the CRC-specific EORTC module EORTC QLQ-CR29, the fatigue-specific EORTC module EORTC QLQ-FA12 (total fatigue and subscores for physical, emotional and cognitive fatigue) and the Patient Health Questionnaire-4 (PHQ-4) (anxiety and depression) are used as validated assessment tools. Questionnaires are handed out at SCR, during every staging visit, at EOT, afterward every 3 months until the EOS and at EOT.
